# Long acting reversible contraceptive utilization and its associated factors among modern contraceptive users in high fertility sub-Saharan Africa countries: a multi-level analysis of recent demographic and health surveys

**DOI:** 10.1186/s13690-022-00977-1

**Published:** 2022-10-24

**Authors:** Wubshet Debebe Negash, Tadele Biresaw Belachew, Desale Bihonegn Asmamaw

**Affiliations:** 1 Department of Health Systems and Policy, Institute of Public Health, College of Medicine and Health Sciences, University of Gondar, Gondar, Ethiopia; 2 Department of Reproductive Health, Institute of Public Health, College of Medicine and Health Sciences, University of Gondar, Gondar, Ethiopia

**Keywords:** Long-acting reversible contraceptive methods, Multilevel, Factors, Sub-Saharan Africa

## Abstract

**Background:**

Long-acting reversible contraceptives (LARC) have been hailed as one of the safest and most effective methods of contraception. However, the use of LARC is low in the world, including Sub Saharan Africa; therefore, the aim of this study was to assess LARC utilization and associated factors among modern contraceptive users in high fertility SSA countries.

**Methods:**

Data for this study was obtained from the most recent Demographic and Health Surveys. A total weighted sample of 14,828 reproductive age women was included. A multilevel mixed-effect binary logistic regression model was fitted to identify the significant associated factors of long acting reversible contraception utilization. Finally, the Adjusted Odds Ratio (AOR) with 95% confidence interval was used to declare as statistical significance.

**Results:**

Overall prevalence of LARC utilization was observed to be 20.1% (19.45, 20.74). The factors significantly associated with the utilization were women’s age ≥ 35 years (AOR = 1.42; 95% CI: 1.19,1.68), having media exposure (AOR = 1.13; 95% CI: 1.05, 1.28), number of alive children: 1–2 (AOR = 2.35; 95% CI: 1.38, 4.01), 3–4 (AOR = 2.98; 95% CI: 1.74, 5.10), $\ge$ 5 (AOR = 2.82; 95% CI:1.63, 4.86), have no history of abortion (AOR = 1.33; 95% CI: 1.17,1.51) and who have no big problem with distance to the health facility (AOR = 1.29; 95% CI: 1.16, 1.43).

**Conclusion:**

The use of long acting reversible contraception in this study was relatively low. To improve the utilization of long acting reversible contraceptives governments, policymakers, and stakeholders should implement health promotion strategies through media and improve accessibilities of health facilities.

## Background

The Family Planning (FP) services provide information, counseling, and birth control methods that help people in making decisions about when and if to have children [1]. It is also the best investment for the health and wellbeing of women, children, and communities in health care [2]. Providing women and girls with affordable, high-quality reproductive health services and information is essential to ensuring their rights and well-being [3].

Contraceptive methods used for family planning can be categorized into modern contraception and traditional methods that are used to limit or postpone childbearing [4]. Modern contraception refers to short-acting, long-acting, and permanent methods of contraception that exclude traditional methods [5, 6]. Long-acting reversible contraception (LARC) has been hailed as one of the safest and most effective methods of contraception [7]. The hormonal contraceptive implant is a reversible, long-acting contraceptive that releases a progestin hormone. Depending on the type, it can provide 3 to 5 years of protection. The intrauterine contraceptive device (IUCD) is also a reversible long-acting contraceptive, which is a small device placed in the uterus to prevent pregnancy [8].

Globally, fewer than 15% of women used LARCs [9]. For example, Europe has a varied prevalence of LARC use; the prevalence of LARC use in Poland is 2.9%, while France has a prevalence of about 16%, which is higher than the global proportion of women using LARCs [10]. Different regions, like Latin America and Asia, have recorded a high prevalence of utilization of LARC [11]. However, in Sub-Saharan Africa (SSA), there was a 3% prevalence of LARC use among women [12]. Low utilization of modern contraceptive methods is a major challenge in most low-resource settings. Due to the low level of LARC method utilization, a large number of maternal deaths are occurring in low and middle-income countries [13]. According to studies conducted in SSA [14–16], 20% of married women in reproductive age use family planning, but less than one in seven also uses long-acting or permanent contraception [14–16]. The low use of reversible long-acting methods of contraception may contribute to an increased number of unintended pregnancies [17, 18]. Due to the factors mentioned above, Sub-Saharan Africa has the highest fertility in the world (5.4 births per woman on average), which is twice higher than that of Asia (excluding China) and more than three times higher than that of Europe [19].

A study conducted in Nigeria found that higher education, over three children, previous LARC use, and the good knowledge and positive attitude of the women about LARC were significant determinants of utilization [20]. The existing studies also revealed that socio-cultural beliefs and practices, level of knowledge, fear of side effects, partner’s objection, convenience, fertility intentions, accessibility, providers’ skill, and competence were determinants of their usage [14, 21]. In addition, different predictors have been identified as being associated with LARC methods. These predictors include socio-demographic aspects, behavioral characteristics, and institutional, and service factors [8, 22].

In countries with high fertility rates and an unmet need for family planning, shifting toward long-acting family planning methods (LAFPMs) is an important strategy to ensure continuity of services [23–25]. However, currently; modern contraceptive use is dominated by short-term methods [26–28]. Even though the utilization of family planning methods in countries with high birth rates and limited resources has the potential for improving maternal and child health, the proportion of users of long-acting contraceptive methods is very low in high fertility countries [29]. Although studies were conducted in specific countries like Nigeria [20], Congo [30], and Burkina Faso [31]. There have not been any studies combined these high fertility countries (Niger, Democratic Republic Congo, Mali, Chad, Angola, Burundi, Nigeria, Gambia, and Burkina Faso).

Despite LARC utilization was conducted in 26 SSA countries [32]; the current study used multilevel analysis to model the hierarchical nature of the data which is differed from the previous study in SSA. In which the former study was not include community level variables such as, community level poverty, distance to the health facility, community level media exposure, and community level education where these variables were incorporated in this study. Moreover, the current study tried to assess additional factors such as number of living children, ever had history of abortion, sex of household head, decision maker for using LARC methods. Evidences revealed that all the aforementioned factors were important to determine LARC utilization [33–36].

Therefore, this study aimed to determine whether LARC is utilized by women in high fertility countries and whether they are associated with other factors. It is hoped that the results of the study will help policymakers to make interventions that will help reduce maternal mortality and morbidity through speeding up the utilization of the LARC method.

## Methods

### Study settings and data source

The study was a cross-sectional assessment of data from Demographic and Health Surveys (DHSs) conducted between January 2010 and December 2018 in high fertility countries in SSA. Countries (Niger, Democratic Republic Congo, Mali, Chad, Angola, Burundi, Nigeria, Gambia, and Burkina Faso) were included in this study. These countries were selected because they are the top ten countries with high fertility rates in SSA with fertility rates above 5.0, a higher value than the rate of 4.44 in SSA and 2.47 worldwide [37]. One country (Somalia) with no DHS data was excluded from the analysis. The data for these countries were obtained from the official database of the DHS program, www.measuredhs.com after authorization was granted via online request by explaining the purpose of our study. We used the woman’s record (IR file) data set and extracted the dependent and independent variables. The DHS is a nationally representative household survey that uses face-to-face interviews on a wide range of population, health, nutrition tracking, and effect assessment measures. Study participants were selected using a two-stage stratified sampling technique. Enumeration Areas (EAs) were randomly selected in the first stage, while households were selected in the second stage. For the sample data to be representative, weighting was conducted before analysis of the DHS dataset since households are not selected uniformly. We used the individual weight for women (v005), which is the household weight (hv005), multiplied by the inverse of the individual response rate. Individual sample weights are generated by dividing (v005) by 1,000,000 before analysis to approximate the number of cases [38, 39]. Finally, a total weighted sample of 14,828 reproductive- age women was included from all nine countries in this study (Table 1).

**Table 1:** Description of Surveys and sample size characteristics in high fertility countries in SSA (n = 14,828)

Countries	Survey year	Weighted sample(n)	Weighted percentage (%)
Angola	2015/16	921	6.2
Burkina Faso	2010	1829	12.3
Burundi	2016/17	2486	16.4
Chad	2014/15	558	3.8
DR Congo	2013/14	2040	13.8
Gambia	2013	483	3.3
Mali	2018	1318	8.9
Nigeria	2012	4052	27.3
Niger	2012	1142	7.7

### Study variables

#### Outcome variable

The outcome variable of this study is long-acting reversible contraceptive use. Long-acting reversible contraceptives include intrauterine device (IUCD) and Implants. Finally, the outcome variable was categorized as yes for those who used one of the above methods, otherwise no and coded as 1 and 0, respectively.

#### Explanatory variables

Both the individual and community level independent variables were included in this study.

**Individual level variables;** age, marital status, educational status, occupation, wealth status, media exposure, number of living children, ever had a terminated pregnancy, husband’s education, Sex of household head, and decision making for using LARC methods.

**Community level variables;** residence, and some of were generated from the individual level data of all community members in primary sampling unit (PSU), which includes the community level poverty, distance to the health facility, and community level media exposure that was defined as the proportion of women who had media exposure in a cluster. The aggregate of individual women’s media exposure can show the overall media exposure of women within the cluster.

### Data analysis

Stata version 16 software was used for data analysis. The data were weighted to ensure the representativeness of the DHS sample and get reliable estimates and standard errors before data analysis.

Four models were fitted in this study: the null model, which had no explanatory variables, model I, which had individual-level factors, model II, which had community-level factors, and model III, which had both individual and community-level components. Since the models were nested, the Intra-class Correlation Coefficient (ICC), Median Odds Ratio (MOR), and, deviance (-2LLR) values were used for model comparison and fitness, respectively. Model III was the best-fitted model since it had the lowest deviance. Variables having a p-value less than 0.2 in bivariable analysis were used for multivariable analysis. Finally, in the multivariable analysis, adjusted odds ratios with 95% confidence intervals and a p-value of less than 0.05 were utilized to identify factors of LARC use.

## Results

### Individual level factors

About 6739 (45.5%) of the women were aged between 25 and 34 years. Majority (83.8%) of the participants were married. Regarding their educational status, 6177 (41.7%) respondents were reported with secondary and above educational levels and 7365 (49.7%) respondents’ husband attained secondary and higher educational levels. Among the participants 5014 (33.8%) had more than three living children. In this study one sixth (17.1%) of the participants experienced abortion. Moreover, the majority of respondents (76.0%) were exposed to media. In addition, 9268 (62.9%) participants jointly made decision with their husbands about LARC utilization. With regard to their economic level, 3502 (23.6%) women were in the poor quintiles and 8854 (59.7%) were in the rich quintiles (Table 2).

**Table 2:** Individual characteristics of respondents in high fertility countries in Sub-Saharan Africa (n = 14,828)

Variables	Category	Weighted number (%)	Weighted prevalence (95% CI)
Age in years	15–24	2779 (18.7)	14.8 (13.5, 16.0)
	25–34	6739 (45.5)	44.5 (42.7, 46.3)
	35+	5310 (35.8)	40.8 (38.9, 42.5)
Sex of household head	Male	13,771 (92.9)	93.6 (92.8, 94.4)
	Female	1057 (7.1)	6.3 (5.6, 7.3)
Current marital status	Married	12,430 (83.8)	90.2 (89.1, 91.2)
	Not married	2398 (16.2)	9.7 (8.8, 10.8)
Educational status of respondents	No education	4994 (33.7)	37.5 (35.8, 39.3)
	Primary education	3658 (24.7)	23.0 (21.6, 24.6)
	Secondary and above	6177 (41.7)	39.3 (37.6, 41.1)
Husband education	No formal	4510 (30.4)	34.5 (32.8, 36.2)
	Primary	2939 (19.8)	20.8 (19.4, 22.3)
	Secondary and higher	7365 (49.7)	44.6 (42.8, 46.4)
Occupation of respondents	Working	10,847 (76.2)	78.2 (76.7, 79.7)
	Not working	3384 (23.8)	21.8 (20.3, 23.3)
Wealth status	Poor	3502 (23.6)	23.3 (21.8, 24.8)
	Middle	2473 (16.7)	17.3 (15.9, 18.7)
	Rich	8854 (59.7)	59.4 (57.6, 61.1)
Media exposure	Yes	11,269 (76.0)	77.5 (76.0, 79.0)
	No	3559 (24.0)	22.5 (20.9, 23.9)
Decision maker for using contraception	Mainly respondents	3733 (25.4)	21.5 (20.0, 22.9)
	Husband	1724 (11.7)	11.8 (10.7, 13.0)
	Jointly	9268 (62.9)	66.7 (64.9, 68.4)
Ever had terminated pregnancy	Yes	2542 (17.1)	14.5 (13.3, 15.8)
	No	12,286 (82.9)	85.5 (84.2, 86.7)
Number of alive children	No	203 (1.4)	0.6 (0.3, 0.9)
	1–2	4709 (31.8)	26.4 (24.8, 27.9)
	3–4	5014 (33.8)	36.5 (34.8, 38.3)
	$\ge$5	4903 (33.1)	36.6 (34.8, 38.3)

### Community level factors

About 7916 (53.4%) of the study participants resided in rural areas. Of the respondents, 7998 (53.9%) were from communities with low proportion poverty level. Majority (70.8%) of respondents had no a big problem with related to health facility distance. More than half (53.0%) of the participants were under high proportion community education level (Table 3).

**Table 3:** Community level characteristics of respondents in high fertility countries in Sub-Saharan Africa (n = 14,828)

Variables	Category	Weighted frequency (%)
Residence	Urban	6912 (46.6)
	Rural	7916 (53.4)
Community-level poverty	Low	7998 (53.9)
	High	6831 (46.1)
Distance to the health facility (n = 14,502)	Big problem	4229 (29.2)
	Not big problem	10,272 (70.8)
Community media exposure	Low	7149 (48.2)
	High	7679 (51.8)
Community education	Low	6971 (47.0)
	High	7857 (53.0)

### Prevalence of long acting contraceptive use

**Fig. 1:**
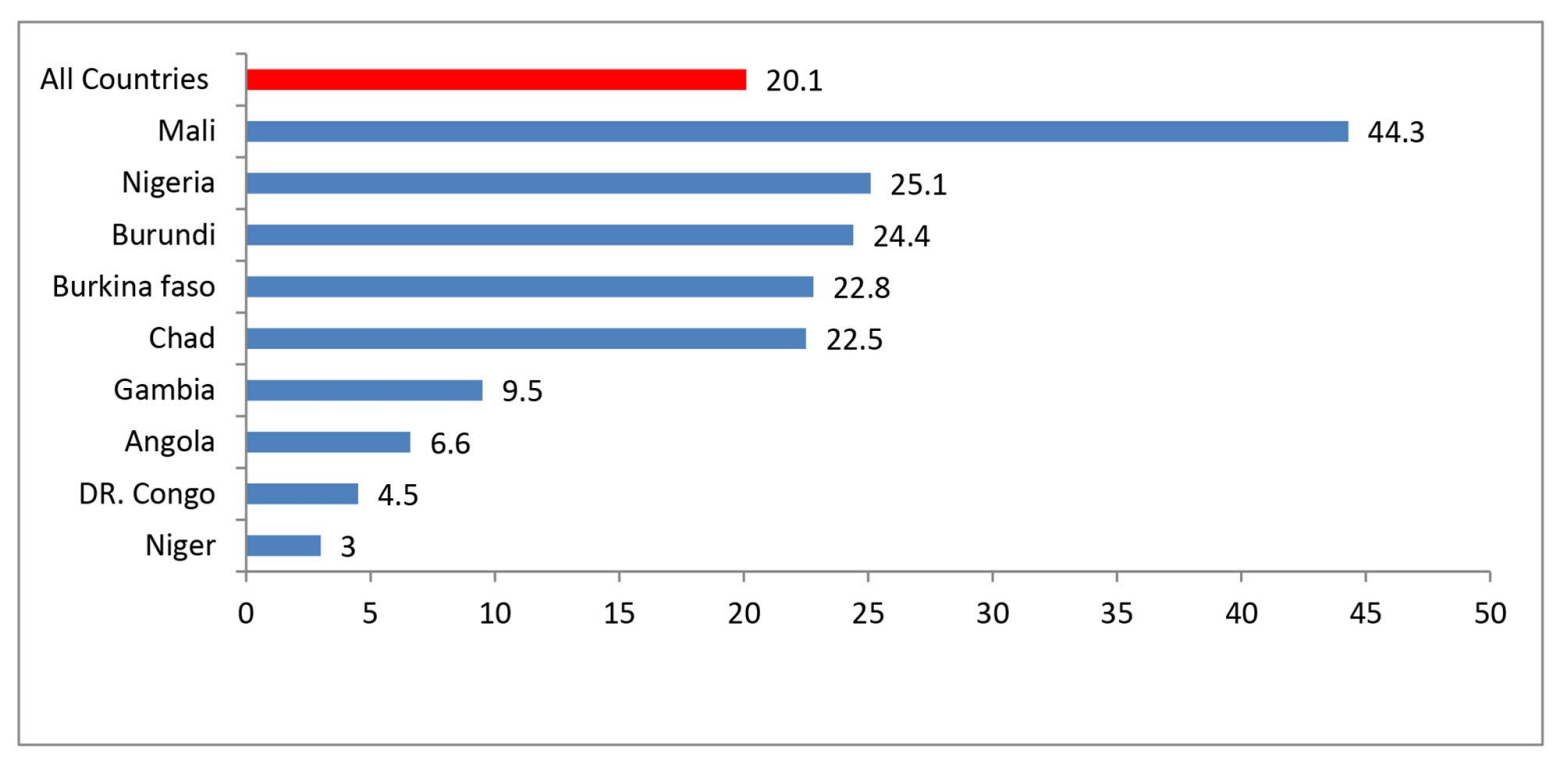
Prevalence of LARC utilization in high fertility SSA countries

Overall, the prevalence of LARC use among reproductive age women in Sub Saran Africa high fertility countries was 20.1% (19.45, 20.74). The LARC use was ranged from 3.0% in Niger to 44.3% in Mali (Fig. 1).

### Factors associated with long acting reversible contraception utilization

From all independent individual and community level factors, maternal age, educational status of women, wealth index, media exposure, number of living children, ever had terminated pregnancy, sex of household head, community level poverty, distance to the health facility, community level media exposure, and community level education were eligible variables for multivariable multilevel analysis. Among eligible individual and community level factors, age, media exposure, number of living children, ever had terminated pregnancy, and distance to the health facility were factors associated with LARC utilization.

Accordingly, individuals aged 35 and above had 1.42 (AOR = 1.42; 95% CI: 1.19, 1.68) higher odds for LARC use compared to those aged 15–24. Individuals exposed to media had 1.13 (AOR = 1.13; 95% CI: 1.05, 1.28) higher odds for LARC use compared to non-exposed. Women who had 1–2 children had 2.35 (AOR = 2.35; 95% CI: 1.38, 4.01) higher odds for LARC use, 3–4 children had 2.98 (AOR = 2.98; 95% CI: 1.74, 5.10) higher odds for LARC use, and 5 + chidren had 2.82 (AOR = 2. 82; 95% CI: 1.63, 4.86) higher odds for LARC use compared to no child. Moreover, individuals who had not terminated pregnancy had 1.33 times higher odds for use of LARC (AOR = 1.33; 95% CI: 1.17, 1.51) than who had terminated pregnancy.

With regard to the community level factors, women who did not have perceived distance to the health facility as not a big problem had 1.29 (AOR = 1.29; 95% CI = 1.16, 1.43) higher odds compared to those perceived as a big problem (Table 4).

**Table 4:** Multivariable analyses for factors affecting LARC use **(n =** 14,828)

Variables	Model 0	Model 1 AOR (95% CI)	Model 2 AOR (95%CI)	Model 3 AOR (95%CI)
Individual level Characteristics				
Age				
15–24		1		1
25–34		1.14(0.99,1.32)		1.12 (0. 97, 1.29)
35+		1.42(1.19,1.67)		1.42 (1.19, 1.68)*
Educational status of the respondents				
No formal education		1		1
Primary education		0.99(0.88,1.12)		0.93 (0.82, 1.05)
Secondary and higher		1.17(1.05,1.32)		1.11 (0.98, 1.26)
Wealth index				
Poor		1		1
Middle		1.02(0.88,1.17)		1.02 (0.88, 1.18)
Rich		1.03(0.91,1.17)		1.13 (0.98, 1.29)
Media exposure				
No		1		1
Yes		1.11(0.98,1.23)		1.13 (1.05, 1.28)*
Sex of household head				
Female		1		1
Male		1.09(0.92,1.30)		1.07 (0.89, 1.28)
Number of alive children				
No child		1		1
1–2		2.32(1.36,3.94)		2.35 (1.38, 4.01)*
3–4		2.95(1.72,5.03)		2.98 (1.74, 5.10)*
≥ 5		2.82(1.64,4.86)		2. 82 (1.63, 4.86)*
Terminated pregnancy				
Yes		1		1
No		1.33(1.18,1.51)		1.33 (1.17, 1.51)*
Community level variables				
Community level poverty				
High			1	1
Low			1.11(0.94,1.32)	1.09 (0.92, 1.29)
Community media exposure				
Low			1	1
High			1.25(1.06,1.46)	1.17 (0.99, 1.38)
Residency				
Rural			8.31(2.88,23.97	1
Urban			0.73 (0.66, 0.89)	0.70 (0.62, 1.02)
Community level education				
High			1	1
Low			1.08 (0.92, 1.27)	1.05 (0.89, 1.25)
Distance to the health facility				
Big problem			1	1
Not big problem			1.29 (1.17, 1.43)	1.29 (1.16, 1.43)*
Random effect result				
Variance (%)	62	51	47	27
ICC (%)	15.67	15.47	15.4	15.3
MOR	20.4	18.6	17.7	13.4
PCV	Ref	17.7	24.2	56.5
Deviance(-2LLR)	14,368	14,228	13,962	13,826

* Statistically significant at p-value < 0.05,

AOR Adjusted Odds Ratio, COR Crude Odds Ratio

Null model: adjusted for individual-level characteristics,

Model 2: Adjusted for community-level characteristics,

Model 3: adjusted for both individual and community level characteristics.

## Discussion

The study was conducted to examine the prevalence of LARC utilization and associated factors among modern contraceptive users in high fertility Sub Saharan Africa countries. According to this study, age of respondents, number of living children, history of terminated pregnancy, media exposure, and distance to the health facility were associated with LARC utilization.

According to this study, only one from five, 20.1% (19.45, 20.74), of reproductive age women uses LARC in high fertility SSA countries. This finding is comparable to those of a studies conducted among reproductive age women in SSA countries with prevalence of (20.73%) [40] and a cross-sectional study conducted in Kenya (20.6%) [41]. The similarities could be attributed to the similar social and political settings of countries in Sub Saharan Africa.

The finding of this study is higher than studies done in Ethiopia (13.1%, 16%) [14, 21], and Nepal 4.7% [34]. However, as the present study, the rate of LARC utilization is lower, compared to Gambia 89% [42], Chad 89% [43], Democratic of Congo 74% [43], and Nigeria 38.7% [44]. The difference can be due to variation in participants’ characteristics and individual and community based nature of our study.

Reproductive women who were ≥ 35 years of age were more likely to use LARCs compared with younger women. The reason could be that they may have enough children up to this age, at which point they will need to keep their family size under control until menopause. This result is supported by a study conducted in Kenya [33]. However, it is differ to a study conducted in Sub Saharan Africa [40].

Women who exposed to media were positively associated with LARC utilization. This result is in line with a study conducted in Ethiopia [44, 45]. A possible explanation is that the media has a powerful ability to explain different methods, their benefits and where they are available to women, enhancing women’s use of the contraceptive methods.

Women who have one to two, three to four, and five and above living children had 2.35, 2.98, and 2.82 times higher odds of LARC utilization respectively, as compared to women who have no living children. This finding is consistent with a study conducted in Ethiopia [46, 47] Uganda [48], Nigeria [49], Malawi [50], Ghana [51]. The possible reasons might be explained as those women who might have already attained their plan of fertility could use LARCs up to reaching menopause or need to space or limit births may choose effective long acting contraceptive methods from the available options.

The odds of using LARC methods were 1.33 times higher among women who did not terminate their pregnancy as compared to those who did. This could be explained by the fact that the individuals had no prior history of abortion and hence could have a sufficient number of children. Therefore, they need to limit the number of their children. This result is different from a study conducted in Ethiopia [52] and a study from Angola reported that women with history of abortion were more likely to use modern family planning compared to women who never had abortion [53].

The odds of using LARC methods were more among women who live near to the health facilities as compared to those who live far from health facilities. The reason might be as the health facility is nearer; the more they have contact time and get information about utilizing LARC, the more likely they use LARC methods.

This study used national representative data, a large sample size, and advanced model to make the results relevant. Because the study was cross-sectional, we could only measure associative rather than causal effects. Due to the fact that we employed secondary data, we were unable to account for certain important variables. It is also important to recognize that this study didn’t examine their beliefs, cultures, or attitudes regarding their use.

## Conclusion

According to this study, the use of long acting reversible contraception in high fertility countries in SSA is relatively low. Age, number of living children, terminated pregnancy, media exposure, and distance to the health facility were independent predictors of LARC use among reproductive-age women in high fertility SSA countries. To improve the utilization of long acting reversible contraceptives governments, policymakers, and stakeholders should implement community and facility-level awareness creation and health promotion strategies through media and improve accessibility of health facilities.

## Data Availability

Data for this study were sourced from Demographic and Health surveys (DHS), which is freely available online at (https://dhsprogram.com).
